# A Comparative In Vitro Study on Heat Generation with Static Guided and Conventional Implant Bed Preparation Using Stainless Steel Twist Drills and a Standardized Bovine Model

**DOI:** 10.3390/ma18061277

**Published:** 2025-03-13

**Authors:** Dino Tur, Zhiwei Tian, Katharina Giannis, Ewald Unger, Martina Mittlboeck, Xiaohui Rausch-Fan, Georg D. Strbac

**Affiliations:** 1 Clinical Division of Periodontology, University Clinic of Dentistry, Medical University of Vienna, 1090 Vienna, Austria; dino.tur@meduniwien.ac.at (D.T.); xiaohui.rausch-fan@meduniwien.ac.at (X.R.-F.); 2 Competence Center for Periodontal Research, University Clinic of Dentistry, Medical University of Vienna, 1090 Vienna, Austria; n11945430@students.meduniwien.ac.at; 3 Clinical Division Unit-Dentistry Training, University Clinic of Dentistry, Medical University of Vienna, 1090 Vienna, Austria; katharina.giannis@meduniwien.ac.at; 4 Center for Medical Physics and Biomedical Engineering, Medical University of Vienna, 1090 Vienna, Austria; ewald.unger@meduniwien.ac.at; 5 Institute of Clinical Biometrics, Center for Medical Data Science, Medical University of Vienna, 1090 Vienna, Austria; martina.mittlboeck@meduniwien.ac.at; 6 Center for Clinical Research, University Clinic of Dentistry, Medical University of Vienna, 1090 Vienna, Austria; 7 Clinical Division of Oral Surgery, University Clinic of Dentistry, Medical University of Vienna, 1090 Vienna, Austria

**Keywords:** guided implant surgery, dental drills, heat generation, thermal osteonecrosis, standardized testing specimens, digital workflow, multiple temperature sensors, surgical templates

## Abstract

The aim of this in vitro study was to evaluate the differences in heat generation across the drilling techniques, depths, and irrigation conditions of static computer-assisted implant surgery (S-CAIS) and conventional implant preparation (CIP) using a standardized bone model for comparative investigation. A total of 240 automated intermittent experimental procedures of 10 and 12 mm drilling depths were performed during S-CAIS and CIP using stainless steel twist drills of three drill diameters (2.2, 2.8, and 3.5 mm) and two irrigation modes (without/external cooling) at room temperature. Temperature changes were recorded in real time using multiple temperature sensors in two distances to the osteotomy site. For comparison, a linear mixed model was fitted. The level of statistical significance was set at α = 0.05. Comparing the two surgical techniques, significant temperature differences could be observed using 3.5 mm drills: CIP yielded statistically higher temperatures during 10 and 12 mm drilling without irrigation (*p* = 0.0115 and *p* = 0.0253, respectively), while statistically higher temperatures were observed with S-CAIS and external irrigation at a 12 mm drilling depth (*p* = 0.0101). This standardized in vitro investigation demonstrated the impact of surgical technique, drilling depth, and irrigation mode on heat generation, indicating differences especially in drills of larger diameter.

## 1. Introduction

Since the first introduction of titanium dental implants more than 50 years ago, implant-supported dental restorations, even in highly complex cases, have become part of the clinical routine [1,2,3,4]. With promising documented outcomes and high primary stability of contemporary threaded dental implants, the indication of treatment is increasingly extended to immediate implant placement and restoration in the esthetic zone [5,6,7,8]. Especially in these clinical situations, tapered implant geometries could be considered a viable alternative to conventional cylindrical implants, as high implant stability quotients and accuracy at the time of placement have been observed [9,10]. Although promising results with tapered implants have been reported over an observation period of 10 years, the longest clinical experience has been gained with cylindrical dental implants over the past decades [11,12,13,14]. Irrespective of the chosen implant geometry, research interest in conventional twist-drill designs for implant preparation remains undimmed, since they are used for the preparation both of tapered and cylindrical implants.

Correct 3-dimensional implant positioning is considered a prerequisite for an ideal esthetic implant restauration, which can be achieved by using a prefabricated surgical guide [15]. In this context, retrospective comparison of implant rehabilitation in the esthetic zone comparing conventional freehand implant placement with a contemporary guided surgical approach yielded superior esthetic outcome in terms of the pink esthetic score with guided procedures after an observation period of 5 years [16]. Further retrospective investigation of different guided surgical techniques during immediate and delayed implantation in the anterior zone resulted in comparable accuracy for both full- and half-guided implant surgery in both investigated implant placement groups, with the highest accuracy in full-guided procedures [17].

The early adoption of denture-based drill guides at the dawn of modern implant dentistry was initially rejected due to feared insufficiency of irrigation of the guided surgical site and an ultimate claim for an atraumatic preparation technique [2]. Excessive surgical trauma is considered to be one of the most common causes for early implant losses and represents failure to establish proper osseointegration [18,19]. From a pathophysiological perspective on a cellular level, cell death due to thermal osteonecrosis may be attributed to protein denaturation, reduced bone cell activity, and dehydration of the injured tissues [20]. As thermal injury to the bone can be a consequence of excessive surgical trauma and tissue temperatures exceeding 44–47 °C with a specific exposure time were found to be critical for the survival of bone, numerous factors contributing to thermal osteonecrosis have been identified ever since [20,21,22]. They include parameters related to the surgical instrument [23,24], the surgical technique [25,26,27], the use of irrigation [28,29], and the surgical site itself [30]. A recent systematic review of the outcome of dental implants placed by means of guided surgery reported early implant failure rates of 1.6% and 3.98% at the implant and patient levels, respectively, with most failures occurring at an early timepoint of healing [31]. Available in vitro research on thermal bone changes with guided implant procedures indicates significantly higher temperature generation when using surgical guides compared to conventional preparation [32,33]. In this regard, attempts to further improve the irrigation efficacy during S-CAIS by modifying the guided surgical design have been recently proposed [34,35]. Previous in vitro investigations in this field, however, have been using a multitude of testing samples of animal or synthetic origin [32,34,36], thereby making the standardization and comparability of obtained testing results difficult. Standardized artificially manufactured testing samples of bovine origin have been proposed for comparative simulation and have been adopted in various investigations ever since [23,28,37,38,39,40,41]. With the increasing clinical use of guided implant systems, this in vitro investigation aims at further investigating the thermal effects of static computer-assisted implant surgery (S-CAIS) in the proposed standardized bovine specimens by implementing the 3 R principles. As previous research on this topic was conducted using cylindrical pilot and twist drills and cylindrical drills are used for preparation with different implant geometries, this study was performed by using sequential twist drills made of stainless steel. The present investigation was based on the working hypothesis that conventional implant preparation would display beneficial temperature effects when compared to S-CAIS. The primary aim of this study was to investigate the thermal effects of S-CAIS in a standardized testing model and thus secondarily to compare different drill diameters and their performance for further improvement of clinical applications.

## 2. Materials and Methods

**Figure 1:**
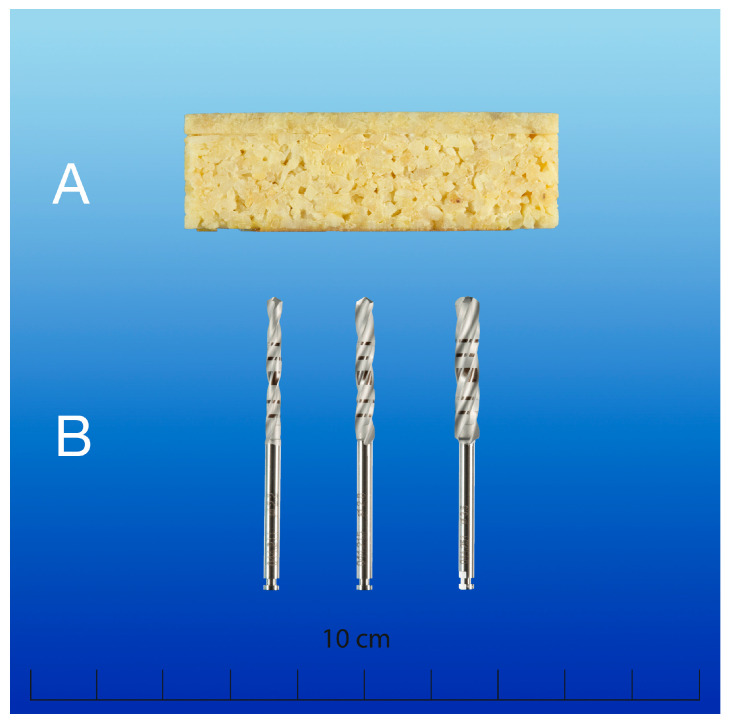
(**A**) Standardized bovine bone specimen (BoneSim^TM^, 1800.35/1300.14 Composite, BoneSim^TM^, Newaygo, MI, USA) with distinctive cortical (3 mm) and cancellous (15 mm) bone sections and (**B**) implant twist drills with respective drill diameters used for the investigation: 2.2, 2.8, 3.5 ∅ (Straumann PRO^TM^, Straumann^®^, Basel, Switzerland).

For the present in vitro study, artificially manufactured specimens of bovine origin (BoneSim^TM^, 1800.35/1300.14 Composite, BoneSim^TM^, Newaygo, MI, USA) were used for simulating guided surgical procedures in a reproducible surgical setting. With human mandibular bone density (type 2 bone according to Lekholm and Zarb classification), the bovine testing samples have been previously suggested for standardized in vitro performance testing of various surgical parameters [23,28,37,38,39,40,41,42,43]. The bone specimens are characterized by thermal conductivity similar to human bone (0.3–0.4 W m^−1^ K^−1^) and distinctive cortical (3 mm) and cancellous (15 mm) bone sections [44]. For simulating mandibular bone sections by means of approximate anatomical proportions of the edentulous alveolar process and for ensuring better simulation of guided surgical preparations, the bone samples were trimmed into a predefined rectangular shape (56 × 18 × 10 mm) prior to the experimental procedure (Figure 1).

Precise and constant width (1 cm ± 0.05 mm) was verified at three predefined measuring points using a digital slide gauge (HSL 246-15; Karl Hammacher GmbH, Solingen, Germany). Commercially available twist drills made of stainless steel for graduated dental implant surgery were used in this investigation (stainless martensitic steel DIN Code: 1.4108; diameters 2.2, 2.8, 3.5 mm; Straumann PRO^TM^, Straumann^®^, Basel, Switzerland) (Figure 1). In accordance with the 3 R principles, this study aimed at further reducing and replacing animal experimentation by using artificially manufactured bone samples; hence, ethics approval was not required for this in vitro investigation.

### 2.1. Digital Preplanning and Manufacturing

**Figure 2:**
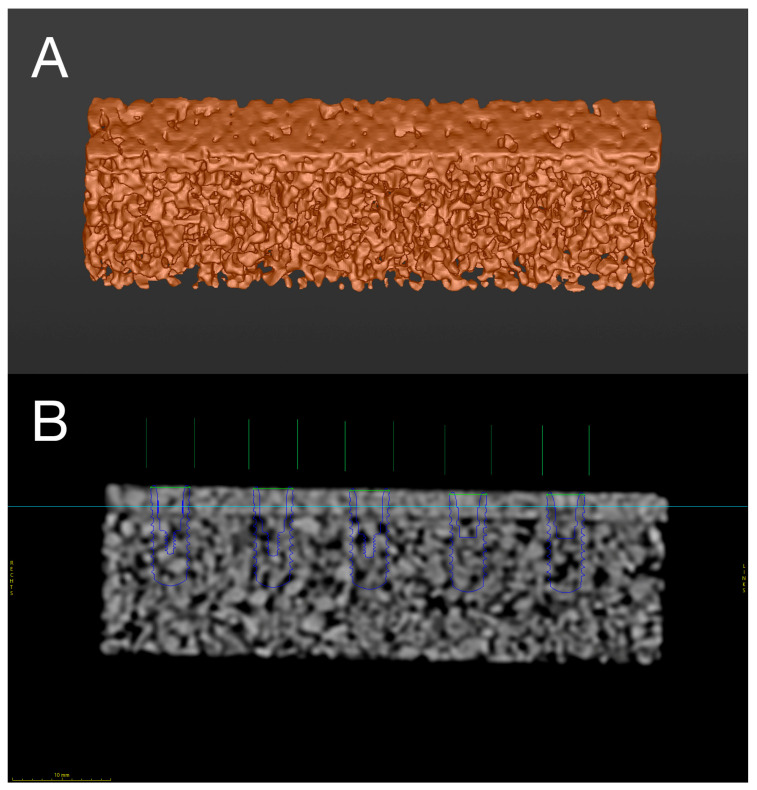
(**A**) Digital Imaging and Communications in Medicine (DICOM) file of bone specimen after subjection to multi-slice computed tomography and (**B**) digital preplanning process including precise implant positioning for experimental surgical procedures, as indicated by the color lines.

Prior to the experimental procedures, the digital preplanning and manufacturing of the surgical guides was performed. To ensure the better in vitro simulation of the clinical situation during static computer-assisted implant surgery (S-CAIS), planning and manufacturing of the surgical guides was performed according to the clinical treatment protocol in guided implant surgery [45]. For the digital planning of the three-dimensional (3D) surgical templates, the bone specimens were subjected to multi-slice computed tomography (MSCT, voxel size 0.2 × 0.2 × 0.5 mm, 120 kV, 140 mAs; Somatom Definition AS; Siemens, Erlangen, Germany) and digital scanning with an intraoral scanner (iTero; Align Technology Inc., San Jose, CA, USA). After obtaining Digital Imaging and Communications in Medicine (DICOM) and segmented stereolithography (STL) files, data were imported into the surgical planning software for guided implant surgery (coDiagnostiX^®^ Version 10.8; DentalWings Inc., Montréal, QC, Canada) (Figure 2).

Five implant preparations per bone specimen were digitally preplanned for the bone level implants of three respective diameters (2.2, 2.8, 3.5 mm; Guided Implant Surgery Bone Level, Straumann^®^, Basel, Switzerland) at two different drilling depths (10 and 12 mm) with an inter-osteotomy distance of 10 mm (Figure 2).

**Figure 3:**
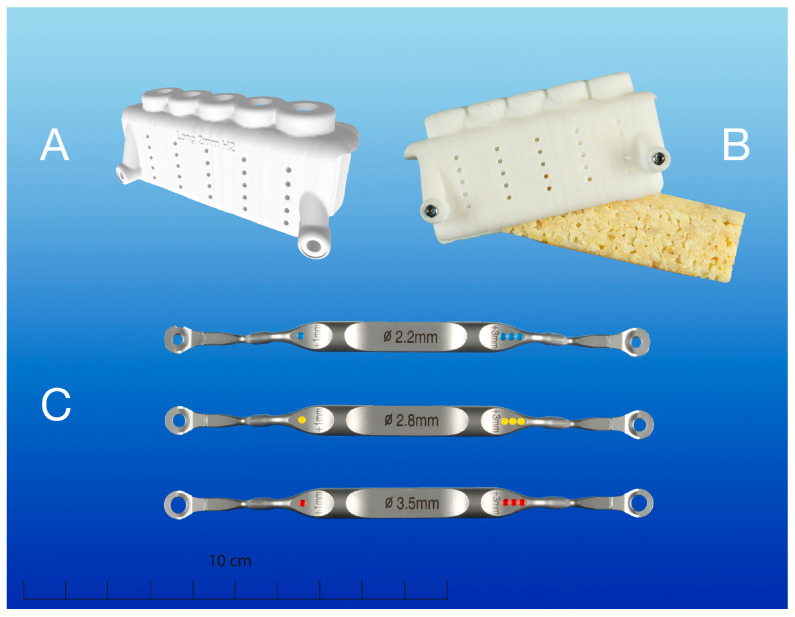
(**A**) Segmented stereolithography (STL) file of the predesigned surgical guide and (**B**) printed surgical guide including two inserted T-sleeves (T-Sleeve for Template Fixation Pin, Ti; Straumann^®^, Basel, Switzerland) with bone specimen before positioning; (**C**) corresponding drill handle cylinders (drill handle, 1 mm/3 mm stop, L 104 mm; diameters 2.2, 2.8 and 3.5 mm, cylinder height 1 mm; Guided Implant Surgery Bone Level, Straumann^®^, Basel, Switzerland).

The surgical guides were digitally predesigned with five sleeveless surgical preparation sites (H2 position) for S-CAIS using the respective drill diameters and corresponding drill handle cylinders (drill handle, 1 mm/3 mm stop, L 104 mm; diameters 2.2, 2.8 and 3.5 mm, cylinder height 1 mm; guided implant surgery bone level, Straumann^®^, Basel, Switzerland) (Figure 3). For the precise positioning of the bone specimens within the surgical guide and for the reproducible preparation of the temperature channels, two T-sleeves (T-Sleeve for Template Fixation Pin, Ti; Straumann^®^, Basel, Switzerland) for insertion of template fixation pins (Template Fixation Pin, diameter 1.3 mm, Ti; Straumann^®^, Basel, Switzerland) were planned at the bottom corners of each guide. Additionally, circular perforations (diameter 1 mm; depths of 3, 6, 9, 12, 15 mm) on both sides of the surgical guide were designed for real-time temperature measurement by inserted temperature sensors. All virtual planning steps were conducted by a senior physician of the Clinical Department of Oral Surgery (University Clinic of Dentistry, Medical University of Vienna, Vienna, Austria). To avoid inaccuracies in the printed guides, the calibration of the preplanned data was performed using 3D-printed calibration matrices. After the completion of the digital preplanning process, data were exported as STL files and printed by a rapid prototyping system (Objet350, Stratasys, Eden Prairie, MN, USA) using an acrylic photopolymer resin (VeroPureWhite^TM^, Stratasys, Eden Prairie, MN, USA) (Figure 3).

### 2.2. Automated Surgical Simulator

For reproducible and standardized surgical procedures, a material testing apparatus (LS1, Lloyd Instruments^TM^, Ametek Inc., Largo, FL, USA) with high-precision vertical displacement was acquired and customized. Exact surgical movement execution was ensured by a wide custom speed range of the apparatus (0.01–2032 mm/min), a minimum load resolution of 0.01 mN, and a total force capacity of 1 kN. Force measurement was performed by a load cell (capacity 250 N, 0.5% accuracy; YLC-0250-A1, Lloyd Instruments^TM^, Ametek Inc., Largo, FL, USA), mounted at the bottom of the crosshead. For external track recording, a draw-wire encoder was used (Kübler D5.3501.A331.0000, draw-wire length transmitter A40/A41, 1 m/10 kΩ potentiometer; Kübler Group, Villingen-Schwenningen, Germany). Simulation of the surgical implant bed preparation was achieved by a software-controlled program (Nexygen^TM^ Plus, Version 4.0, Lloyd Instruments^TM^, Ametek Inc., Largo, FL, USA), allowing for the individual programming of intermittent vertical movements. Two drilling sequences for atraumatic surgical preparation with constant drilling and withdrawing feed rate, with depth control and dwell time having been predefined for both preparation depths, as suggested in previous investigations [23,28,29,40,41]. The 10 mm drilling was programmed with 31.5 s (drilling time 20.6 s, withdrawing time 10.9 s) and 12 mm drilling with 38.9 s (drilling time 26 s, withdrawing time 12.9 s). The feed rate for active preparation was set to 2 mm/s in cortical and subcortical bone sections, 1 mm/s in deeper layers of bone, and 1 mm/s for all intermittent and final withdrawing movements. The locating dowel of the testing apparatus was equipped with a surgical handpiece (WS-75 E/KM 20:1, W&H, Bürmoos, Austria) via a 3D-printed acrylic clamp. A separate customary surgical motor unit (Implantmed SI-923; Surgical control S-N1, W&H, Bürmoos, Austria) was used for operating the surgical handpiece, ensuring external control of drilling parameters and irrigation.

### 2.3. Temperature Measurement

**Figure 4:**
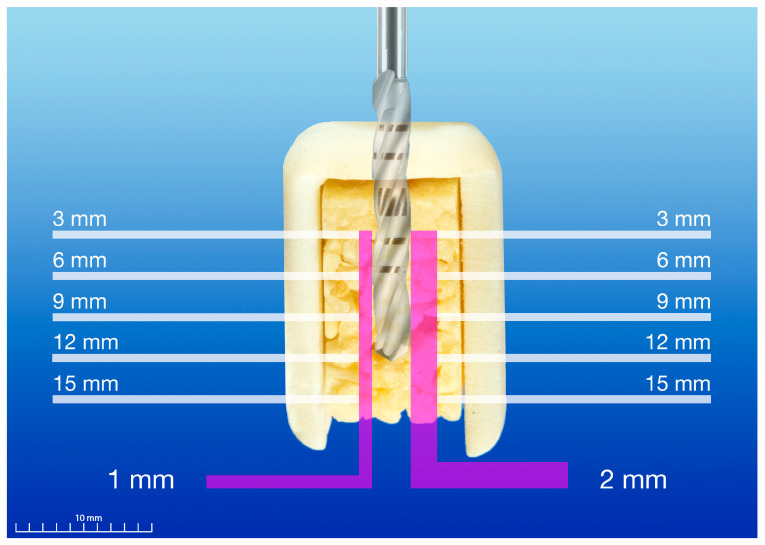
Schematic illustration of real-time temperature measurement system during experimental static computer-assisted implant surgery: 10 individual temperature sensors and their respective measurement depths at distances of 1 and 2 mm from the drilling site.

**Figure 5:**
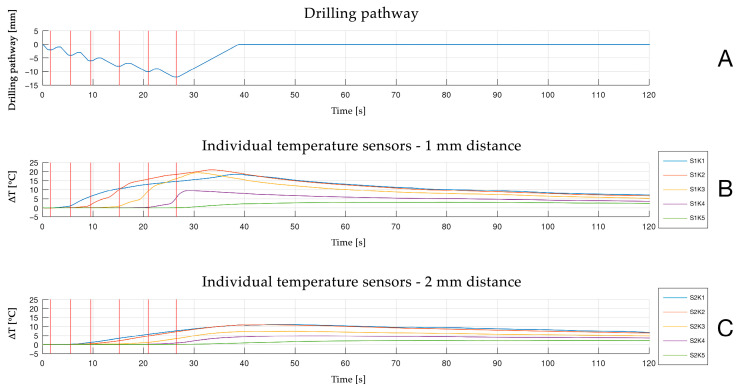
Illustration of multichannel real-time measurement of temperature changes (∆T) at a 12 mm drilling depth with (**A**) drilling pathway recorded by external linear motion potentiometer in (**B**) 1 and (**C**) 2 mm distance to the osteotomy site; red lines indicating corresponding intermittent drilling steps during temperature measurement.

Constant temperature measurement in two radial distances from the final drilling site (1 and 2 mm) and 5 respective measurement depths (3, 6, 9, 12 and 15 mm depth) was enabled by individual real-time temperature sensors (RS PRO Type K Thermocouple, 1 m length, 0.076 mm diameter, +260 °C, RS 397-1589; RS Components Ltd., Corby, UK), based on type-K thermoelements with a material-based thermovoltage (Figure 4). Measurement distances of 1 and 2 mm to the preparation site were chosen for a better comparison of testing results with similar investigations in the past [23,28,29,40,41]. Due to exact temperature measurement by means of individual temperature sensors, a minimum distance of 1 mm to the drilling site was required for the protection of the sensors. Hence, even higher temperatures may be clinically expected at the osteotomy site. For data acquisition, a multifunction measurement and control module (USB-2416 24-Bit, 1 KS/s, Temperature and Voltage Device; Measurement Computing^TM^ Corp., Norton, MA, USA) and a data acquisition software (DAQami, Version 4.2.1f0; Measurement Computing^TM^ Corp., Norton, MA, USA) were used (Figure 5). Before conducting the experimental procedure, preparation steps for temperature measurement were performed: Custom-made sensor arrays were manufactured (vertical distance between temperature channels 3 mm; Center for Medical Physics and Biomedical Engineering, Medical University of Vienna, Vienna, Austria) by means of 3D-printed design and manufacturing. Five individual temperature sensors were bonded into the body of each sensor array by using a light-curing adhesive (Bondic BC5000; Viko UG, Kranzberg, Germany), ensuring a precise and equidistant position of the individual temperature sensors within the bone specimens.

For temperature measurement, the bone samples were prepared prior to the experiment: the precise predrilling of the temperature channels and a reproducible positioning of the surgical guide during the experiment was ensured by pin fixation at the bottom corners of the bone sample according to the previous digital preplanning steps by using a drill for template fixation (Drill for Template Fixation Pin, diameter 1.3 mm, stainless steel; Straumann^®^, Basel, Switzerland). The following predrilling steps of the individual temperature channels were ensured using 3D-printed acrylic guides with preplanned circular perforations by means of a stationary drilling machine (TBM 220; Proxxon S.A., Wecker, Luxemburg) and preparation by multi-twist drills (1.2 mm × 38 mm × 16 mm 118°, HK 11037101, U4 HSSE TiNAlOX 5xD, DIN 338; Atorn^®^, Sartorius Werkzeuge GmbH & Co. KG, Ratingen, Germany). In order to obtain high precision during predrilling for the temperature channels, new 3D-printed acrylic guides were used for each predrilling cycle. For the exact depth positioning of the sensor array at distances of 1 and 2 mm from the future preparation site, stainless steel sleeves (diameter 1.3 mm) were welded to the multi twist drills for depth control during temperature channel preparation. The application of a heat transfer compound (RS Heat Sink Compound Plus, thermal conductivity 2.9 W/(mK), RS 217-3835; RS Componnents Ltd., Corby, UK) in the individual temperature channels was used to obtain constant thermal conductivity during temperature measurement [23,27,28,29,40,41,46]. After inserting the heat transfer compound, the surgical guides were placed on the bone samples and fixed by means of two fixation pins. The testing of conventional drilling was performed by temperature measurement without using drill guides. Real-time data were recorded from 10 s before drilling to 55 s after drilling was concluded.

### 2.4. Experimental Protocol

For this in vitro investigation, a total of 48 rectangular bone specimens have been used. The experimental protocol consisted of 3 drill diameters (2.2, 2.8, 3.5 mm), 2 drilling depths (10 and 12 mm), 2 irrigation methods (with and without external irrigation) and 2 surgical techniques (conventional and static computer-assisted implant surgery). For each constellation, 10 identical repetitions were performed by means of new and unused implant drills for each preparation (*n* = 240 preparations in total). As previously suggested, the drilling speed was set at 800 rpm for a standard atraumatic preparation protocol [23,28,29,40,41,46,47,48,49]. The experimental procedures were conducted under constant room temperature (21 ± 1 °C) in the experimental laboratory for the investigated bone samples. S-CAISs were performed using corresponding drill handle cylinders with a 1 mm cylinder height. For externally irrigated testing procedures, saline irrigation (Ecobag^®^ click, 0.9% NaCl, 5000 mL, B. Braun Melsungen AG, Melsungen, Germany) at room temperature with a constant flow rate of 50 mL/min was used throughout the entire surgical preparation supplied by an irrigation tubing set (Irrigation set for machinery—80 mm, 32.F0139, Omnia^®^, Fidenza, Italy). Surgical suction was ensured at a constant distance (1.5 cm) from the preparation site. Predrilling to the respective previous drilling diameter for an incremental, graduated drilling protocol was performed prior to the experiment for all 2.8 and 3.5 mm preparations. To avoid possible bias during the experimental protocol due to manual handling, the samples were held in place in a vise, and mounted to the lower anchor pin of the automated apparatus. Potential bias due to drill wear was consciously excluded by only using new out-of-the-box instruments. In case of elevated room temperature (temperature > 22 °C), experimental drilling was suspended for the day. The experimental procedures were conducted during the years 2023 and 2024.

### 2.5. Statistical Analysis

Data recording was performed separately for each experimental osteotomy, containing real-time recordings (δ = 0.01 s) of temperature (°C), extension (mm), and data from an external linear measurement device (Draw-wire encoder A40, potentiometer output 10 kΩ; Fritz Kübler GmbH, Villingen-Schwenningen, Germany) (V). For avoiding potential bias and for the comparison of testing results with previous research in this field, temperature changes were calculated [ΔT(°C) = Tx − T0] for subsequent statistical analysis by subtracting the recorded absolute temperature [Tx] from the initial baseline temperature [T0] [23,27,28,29,37,40,41,46,50,51]. Temperature increases were normally distributed and thus described by the means of ±standard deviations. Depth locations of maximum temperature increases were described by median, minimum, and maximum. For a comparison of the temperature increase between surgical preparation techniques, a linear mixed model was fitted including the variables technique (conventional and static computer-assisted implant surgery), drilling diameter (2.2, 2.8, 3.5 mm), drilling depth (10 mm, 12 mm), and irrigation (external, without) together with their interactions up to a four-way interaction term. Adjustments for unequal variances were performed by the degrees-of-freedom adjustment of Kenward–Roger. Assumptions of normally distributed residuals were assessed graphically. In cases of significant interactions, subgroup analyses to test for differences in the surgical techniques were performed by t-tests with unequal variances. Statistical calculations were performed with the statistical software SAS^®^ (Version 9.4, SAS Institute Inc., Cary, NC, USA). All *p*-values are two-sided, and *p* ≤ 0.05 was considered statistically significant.

## 3. Results

A total of 240 experimental procedures were performed for investigating the thermal changes of static computer-assisted implant surgery (S-CAIS) and conventional implant preparations (CIPs) using the drills of three diameters (2.2, 2.8, 3.5 mm). The in vitro experiments included two irrigation modes (without irrigation, external irrigation) and two drilling depths (10, 12 mm). According to the investigated subgroups, 120 preparations were conducted for each drilling depth, irrigation mode, and surgical technique. Eighty preparations were conducted for each investigated drill diameter.

### 3.1. Maximum Temperature Increase

**Figure 6:**
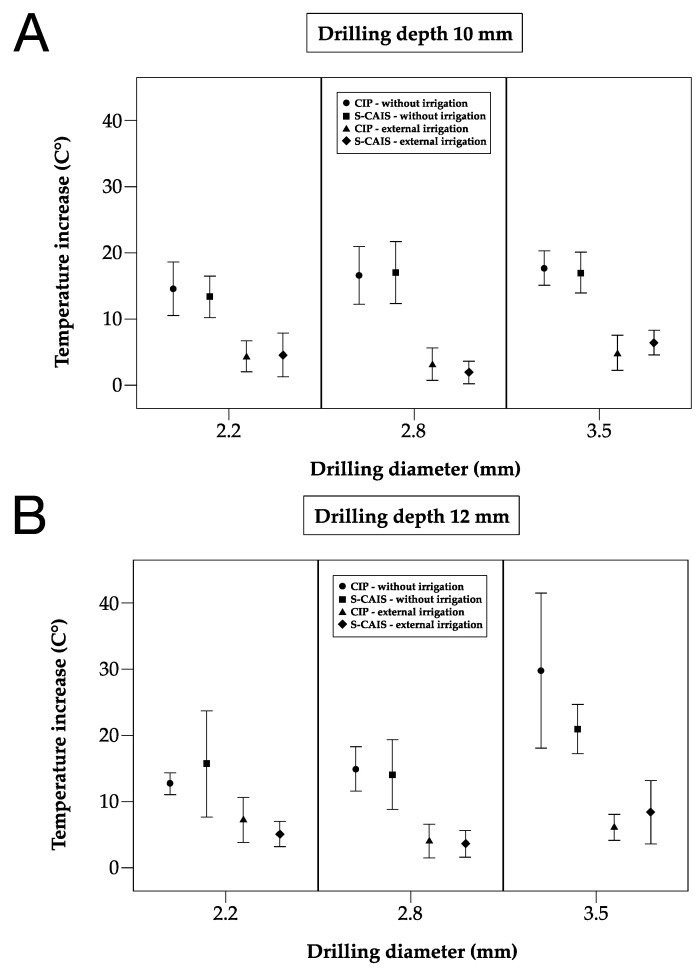
Temperature increase in investigated surgical techniques of different drill diameters (2.0/2.2, 2.8, 3.5 ∅), irrigation methods (without/external irrigation) at (**A**) 10 mm drilling depth and (**B**) 12 mm drilling depth.

The maximum temperature increase over all temperature sensors (2 × 5 sensors) per drill is described for the 10 repetitions in Table 1 by mean values and standard deviations [∆T°C mean (SD)] and with respective temperature results in Figure 6.

The means of the maximum temperature increases for 10 mm drilling sequences without irrigation were as follows [∆T°C mean (SD)]: 13.06 (2.03) for S-CAIS and 13.62 (2.93) for CIP using 2.2 mm drills; 16.70 (3.04) for S-CAIS and 16.54 (3.01) for CIP using 2.8 mm drills; and 15.05 (1.90) for S-CAIS and 17.36 (1.76) for CIP using 3.5 mm drills (Table 1).

The means of the maximum temperature increases for 10 mm drilling sequences with external irrigation were as follows [∆T°C mean (SD)]: 3.78 (1.86) for S-CAIS and 3.86 (1.38) for CIP using 2.2 mm drills; 2.14 (1.12) for S-CAIS and 2.44 (1.39) for CIP using 2.8-mm drills; and 6.36 (1.15) for S-CAIS and 5.25 (1.90) for CIP using 3.5 mm drills (Table 1).

The means of the maximum temperature increases for 12 mm drilling sequences without irrigation were as follows [∆T°C mean (SD)]: 15.75 (4.91) for S-CAIS and 12.86 (1.15) for CIP using 2.2 mm drills; 13.82 (4.22) for S-CAIS and 15.08 (2.20) for CIP using 2.8 mm drills; and 20.80 (2.66) for S-CAIS and 27.10 (7.27) for CIP using 3.5 mm drills (Table 1).

The means of the maximum temperature increases for 12 mm drilling sequences with external irrigation were as follows [∆T°C mean (SD)]: 5.29 (1.05) for S-CAIS and 5.93 (2.08) for CIP using 2.2 mm drills; 3.39 (1.50) for S-CAIS and 4.13 (1.69) for CIP using 2.8 mm drills; and 9.14 (3.00) for S-CAIS and 6.00 (1.27) for CIP using 3.5 mm drills (Table 1).

### 3.2. Temperature Increase and Surgical Technique

Surgical preparation techniques (S-CAIS vs. CIP) were compared in a mixed linear regression model adjusted for irrigation, drilling depth and drilling diameter and also investigating interactions. As the four-way interaction was statistically significant (*p* = 0.0135), indicating that mean differences in the maximum temperature increase depend on irrigation, drilling depth, and drilling diameter, subgroup analyses were performed (Table 1, Figure 6).

Statistically significant differences were only observed with 3.5 mm drills but not with drills with smaller diameters. During the 10 mm preparation depth, 3.5 mm drills, and without irrigation, significantly higher mean temperature differences were observed with CIP compared to S-CAIS (*p* = 0.0115) (Table 1, Figure 6). During 12 mm osteotomy depth and 3.5 mm drills, significantly higher mean temperature differences were observed with CIP without irrigation (*p* = 0.0253) and with S-CAIS with external irrigation (*p* = 0.0101) (Table 1, Figure 6).

### 3.3. Temperature Increase and Sensor Location/Corresponding Depth

For the analysis of temperature distribution within the bone sample, the occurrence of maximum temperature changes at the median sensor channel depth (including minimum and maximum) for the investigated sensor channel depths of 3, 6, 9, 12, and 15 mm was calculated for 1 and 2 mm temperature measurement distances in all investigated constellations (Table 2).

#### 3.3.1. Drill Diameter 2.2 mm

Maximum temperature changes with 2.2 mm drills during 10 mm drilling sequences were observed between median sensor channels of 3 and 6 mm without irrigation and 6 and 9 mm with external irrigation (Table 2). Highest temperature changes with 2.2 mm drills during 12 mm drilling sequences were observed between median sensor channels of 4.5 and 6 mm without irrigation and 9 and 12 mm with external irrigation (Table 2).

#### 3.3.2. Drill Diameter 2.8 mm

Maximum temperature changes with 2.8 mm drills during 10 mm drilling sequences were observed between median sensor channels of 3 and 6 mm without irrigation and 7.5 and 9 mm with external irrigation (Table 2). The highest temperature changes with 2.8 mm drills during 12 mm drilling sequences were observed between median sensor channels of 3 and 6 mm without irrigation and 9 and 12 mm with external irrigation (Table 2).

#### 3.3.3. Drill Diameter 3.5 mm

Maximum temperature changes with 3.5 mm drills during 10 mm drilling sequences were observed between the median sensor channels of 3 and 6 mm without irrigation and 6 and 9 mm with external irrigation (Table 2). The highest temperature changes with 3.5-mm drills during 12 mm drilling sequences were observed between the median sensor channels of 3 and 6 mm without irrigation and 7.5 and 9 mm with external irrigation (Table 2).

## 4. Discussion

In this in vitro investigation, the thermal effects of conventional (CIP) and guided implant preparation (S-CAIS) were investigated by means of a standardized bovine bone sample and automated, intermittent surgical procedures. The simulation of a surgical intervention in a standardized in vitro setting was attempted by using a digital workflow that included preplanning and manufacturing steps, used in contemporary implantology [52,53]. The results of this investigation were partially in alignment with previous studies in the field, reporting higher temperatures with S-CAIS when compared to CIP [32,33,54,55,56]. However, significant temperature differences were not observed with all investigated drill diameters, but were mainly recorded with drills of larger width.

Today, static guided implant surgery is described with a high accuracy regarding implant position [57] and yields promising and reliable results by means of systematic review and meta-analysis [58]. Early concerns raised by Brånemark et al. regarding the use of surgical drill guides fixed to denture base plates were based on the feared inefficiency of irrigation and subsequent implant failure [2]. From a physical point of view, the drilling energy of reaming and shearing processes is mostly converted into heat during bone preparation. Parameters including the quantity of generated heat, the position of the heat source and the heat exposure time, as well as material- and tissue-based parameters including thermal conductivity and heat dissipation characteristics, are considered to be incremental for the heat generation process [59]. With that said, S-CAIS is shown to be associated with additional challenges in terms of heat generation, when compared to CIP: The use of a surgical guide and incorporated sleeves is described as a thermal disadvantage by inducing higher frictional forces due to additional contact between the drill and the sleeves during the preparation process on the one hand and by additionally shielding the preparation site from the irrigation fluid, on the other hand [60].

Previous studies comparing CIP vs. S-CAIS predominantly yielded a higher temperatures with S-CAIS [33,54,55,56], with some authors reporting the highest temperature generation in the initial pilot drills [32]. Substantial temperature differences regarding specific surgical instrument designs were observed for S-CAIS. Thereby, drill geometry seems to significantly influence the amount of heat generation during S-CAIS, especially in drills of smaller diameters [61]. Additionally, previous results in this field of research have been obtained using a magnitude of different samples and materials for testing [32,34,35]. The introduced standardized testing samples of bovine origin are, in contrast, providing similar human mandibular bone density with distinctive and therefore constant cortical and cancellous bone sections throughout the entire sample [28]. For a better simulation of the anatomic properties of mandibular alveolar bone during S-CAIS, samples of a rectangular shape have been proposed in the present study. The chosen experimental setup is therefore aiming for the higher standardization of in vitro testing procedures and better comparison of the obtained results.

In the present investigation, significantly higher temperature differences could only be observed during S-CAIS in 12 mm drilling sites and with drills of 3.5 mm diameter using external irrigation, when compared to CIP. The investigated drill diameters of 2.2 and 2.8 mm with external irrigation did not reveal significantly different results between S-CAIS and CIP and yielded lower mean temperature differences when compared to 3.5-mm diameter drills. These results seem to be in contrast to previous in vitro findings suggesting the highest temperature generation to be found with the smallest drill diameter within the incremental drilling protocol [23,32,62]. A possible explanation for this observation was offered by Frösch et al., who encountered similar trends by investigating the temperature generation during CIP and S-CAIS: An observed higher temperature difference between the drills of large diameter compared to narrow pilot drills during CIP and S-CAIS was believed to be attributed to the size of the flutes and the resulting irrigation efficiency. It was suggested that the irrigation of increased drill diameters with larger drill flutes was more likely to be impaired by the use of a surgical guide when compared to smaller drills [32]. These considerations and the fact that a constant drilling speed of 800 rpm was applied with all the drill diameters in our investigation rather than reduced drilling speeds for larger drills may partially explain the observed results of the present study.

Overall, the cooling efficiency of S-CAIS is described to be substantially influenced by multiple contributing factors, including specific design features of the surgical guide itself and the design of the guiding sleeves [35,63,64,65,66]. On a cellular level, beneficial results by means of immunohistochemical analysis have been reported for CIP when compared to guided implant surgery, indicating earlier bone resorption in the guided group [67]. Additionally, significant differences in the mean bone levels were reported in implants placed by means of different guided surgical techniques, indicating multifactorial biological and clinical influencing factors [58]. Since the present investigation was focusing on 10 and 12 mm preparations only, even higher temperatures may be expected with increased drilling depths and the limited irrigation capacity of external cooling. This might be of particular interest for zygomatic implant placement, which is increasingly used in highly compromised maxillary bone and thereby yielding promising clinical results even in immediate loading [68,69]. Attempts to further optimize the irrigation efficiency during S-CAIS led to the introduction of additional cooling strategies during guided surgical procedures: However, the use of supplementary internal irrigation systems was not found to be advantageous for heat reduction in vitro, when compared to external irrigation alone [70]. Clinically, the application of additional cooling strategies with S-CAIS was already described in multiple case reports [71,72]. The data of our investigation were able to confirm that external irrigation alone is an effective surgical method for reducing intrabony temperatures during guided surgery [56,73]. Substantial heat reduction was observed in all investigated constellations by adding external irrigation, regardless of the drill diameter or the surgical technique. Regarding the defined critical threshold for the survival of bone, the observed temperatures were well below 47 °C with both surgical techniques, provided that external irrigation was used [21]. Additionally, the depth of maximum temperature generation was shown to invariably shift into deeper layers of bone during external irrigation, confirming a substantial cooling effect of external irrigation in superficial bone layers [62], even during S-CAIS [56]. Since room temperature saline irrigation was used in this experiment, additional heat reduction may be assumed with refrigerated saline fluid [33,74].

Lately, research in contemporary implantology has focused on treatment concepts and outcomes for short dental implants. High survival rates for 4 mm implants were recently reported with up to 10 years of follow-up, providing a promising treatment option for patients with vertically reduced bone height [75]. Systematic review and meta-analysis of short (≤6 mm) vs. conventional (≥10 mm) dental implants found short implants to be a viable alternative for implant placement in both jaws in native bone, based on evidence of 5-year RCTs [76]. These considerations inspired the design of the present investigation, focusing on a shorter implant preparation protocol of 10 and 12 mm drilling depths. Thereby, increased mean temperature differences could be almost invariably observed with 12 mm osteotomy depth, confirming a higher temperature generation with deeper preparation depths and longer exposure time to the drilling process [20,23,28,29,41,77]. The temperature measurement protocol of this in vitro investigation is in line with previous studies in the field, providing real-time temperature data in variable depths and distances to the osteotomy site [23,28,29,39,55,56]. Some investigations on CIP vs. S-CAIS used infrared thermography instead, thereby aiming for an overall thermal profile of the surgical area [32,33]. However, doubts have been expressed in the past with this temperature recording method regarding the accuracy using saline irrigation [78,79].

This investigation had some limitations. Live tissues are characterized by human body temperature and constant blood flow, which could not be simulated in a comparable and reproducible in-vitro setting. It may therefore be assumed that in vivo temperatures could vary from the observed results in vitro. Since this study aimed for standardized and comparable research results, no direct simulation of human body conditions was attempted in the experiments. Preparations were rather performed at room temperature for reproducible and comparable testing results. Furthermore, actual temperatures may vary in vivo due to the properties of vital tissues. Consequently, the generalizability of the testing results presented is limited to the comparative trends of the investigated parameters. Since only closed guided procedures were investigated, thermal effects with other types of guides may vary. The implications of the obtained results for clinical practice may include the overall positive effect of external irrigation during S-CAIS, although the cooling efficiency of larger diameters during S-CAIS seems to be less effective and thus needs to be considered with careful attention. Future studies in this field of research may focus on further investigating specific factors contributing to heat generation during S-CAIS. Additionally, other drills and preparation protocols, including drills of larger diameters, should be included in the experiments.

To the best knowledge of the authors, this study can be considered the first in vitro investigation focusing on heat generation by using a standardized bovine bone model and an automated testing apparatus for atraumatic surgical simulation.

## 5. Conclusions

This standardized in vitro investigation has demonstrated the impact of static guided surgical procedures on heat generation by utilizing an automated and atraumatic preparation protocol in standardized bone samples. External irrigation was shown to yield a substantial heat reduction, irrespective of surgical technique and drill diameter. Surgical drills of 3.5-mm diameter exerted the highest mean temperature increase during bone preparation and external irrigation in both surgical techniques, indicating the importance of sufficient temperature control, even in the graduated preparation of larger diameters. The present in vitro results may therefore further contribute to patient safety and improved long-term implant success in the age of digital implantology.

## Figures and Tables

**Table 1:** Comparison of maximum temperature increase: Mean and standard deviation of [∆T°C mean (SD)] of the maximum temperature increase over all temperature sensors at different drilling depths with various drill diameters and irrigation methods, testing static computer-assisted implant surgery (S-CAIS) versus conventional implant preparation (CIP); *p*-values for the subgroups are calculated by a t-test with unequal variances.

Diameter	Technique/*p*-Value	Drilling Depth 10 mm		Drilling Depth 12 mm	
		Without Irrigation	External Irrigation	Without Irrigation	External Irrigation
2.2 mm	S-CAIS	13.06 (2.03)	3.78 (1.86)	15.75 (4.91)	5.29 (1.05)
	CIP	13.62 (2.93)	3.68 (1.38)	12.86 (1.15)	5.93 (2.08)
	*p*-value	0.6228	0.8934	0.1004	0.3942
2.8 mm	S-CAIS	16.70 (3.04)	2.14 (1.12)	13.82 (4.22)	3.39 (1.50)
	CIP	16.54 (3.01)	2.44 (1.39)	15.08 (2.20)	4.13 (1.69)
	*p*-value	0.9051	0.6005	0.4156	0.3172
3.5 mm	S-CAIS	15.05 (1.90)	6.36 (1.15)	20.80 (2.66)	9.14 (3.00)
	CIP	17.36 (1.76)	5.25 (1.90)	27.10 (7.27)	6.00 (1.27)
	*p*-value	0.0115 *	0.1342	0.0253 *	0.0101 *

* *p* ≤ 0.05.

**Table 2:** Depth location of maximum temperature generation: Location of maximum temperature increase: sensor channel location [median, minimum, maximum] (sensor channel depths: 3, 6, 9, 12, 15 mm) in 1 and 2 mm measuring distance at different drilling depths with various drill diameters and irrigation methods (S-CAIS = static computer-assisted implant surgery, CIP = conventional implant preparation).

Diameter	Technique	Distance	Drilling Depth 10 mm						Drilling Depth 12 mm					
			Without Irrigation			External Irrigation			Without Irrigation			External Irrigation		
			Med	Min	Max	Med	Min	Max	Med	Min	Max	Med	Min	Max
2.2 mm	S-CAIS	1 mm	6	3	6	9	6	9	6	6	6	12	6	12
		2 mm	3	3	6	9	6	9	6	3	6	9	6	12
	CIP	1 mm	6	3	9	9	6	9	6	6	9	12	9	12
		2 mm	3	3	6	6	6	9	4.5	3	9	12	6	15
2.8 mm	S-CAIS	1 mm	6	3	6	7.5	3	12	6	3	9	11	6	12
		2 mm	3	3	6	7.5	3	15	6	3	6	9	6	12
	CIP	1 mm	6	3	9	9	9	12	6	3	9	12	6	12
		2 mm	4.5	3	6	9	6	15	3	3	6	12	6	12
3.5 mm	S-CAIS	1 mm	3	3	6	9	3	9	6	3	6	9	9	12
		2 mm	3	3	6	6	6	9	6	3	6	7.5	6	12
	CIP	1 mm	3	3	6	9	6	9	6	3	6	9	6	12
		2 mm	6	3	6	6	6	9	3	3	6	9	6	12

## Data Availability

The original contributions presented in this study are included in the article/Supplementary Material. Further inquiries can be directed to the corresponding author.

## Supplementary Materials

The following supporting information can be downloaded at: https://www.mdpi.com/article/10.3390/ma18061277/s1, Supplementary Materials file: STROBE Statement—checklist of items that should be included in reports of observational studies.

## Abbreviations

The following abbreviations are used in this manuscript:

3D	three-dimensional
CIP	conventional implant preparation
DICOM	Digital Imaging and Communications in Medicine
MSCT	multi-slice computed tomography
RCT	randomized controlled trial
S-CAIS	static computer-assisted implant surgery
SD	standard deviations
STL	segmented stereolithography
